# Validation of the Greek version of the Problematic Internet Use Questionnaire - Short Form (PIUQ-SF-6)

**DOI:** 10.14806/ej.26.1.978

**Published:** 2021-08-23

**Authors:** Panagoula Aivali, Vasiliki Efthymiou, Artemis K. Tsitsika, Dimitrios Vlachakis, George P. Chrousos, Christina Kanaka-Gantenbein, Flora Bacopoulou

**Affiliations:** 1 Postgraduate Course of Science of Stress and Health Promotion, School of Medicine, National and Kapodistrian University of Athens, Athens, Greece; 2 University Research Institute of Maternal and Child Health & Precision Medicine and UNESCO Chair on Adolescent Health Care, National and Kapodistrian University of Athens, Aghia Sophia Children’s Hospital, Athens, Greece; 3 Adolescent Health Unit, Second Department of Pediatrics, School of Medicine, National and Kapodistrian University of Athens, “P. & A. Kyriakou” Children’s Hospital, Athens, Greece; 4 Laboratory of Genetics, Department of Biotechnology, School of Applied Biology and Biotechnology, Agricultural University of Athens, Athens, Greece; 5 Lab of Molecular Endocrinology, Center of Clinical, Experimental Surgery and Translational Research, Biomedical Research Foundation of the Academy of Athens, Athens, Greece; 6 Division of Endocrinology, Diabetes and Metabolism, Center for Rare Pediatric Endocrine Diseases, First Department of Pediatrics, School of Medicine, National and Kapodistrian University of Athens, Aghia Sophia Children’s Hospital, Athens, Greece

## Abstract

Internet is a necessary tool of everyday routine, however, concerns about the development of abnormal behaviours in relation to its use by adolescents are constantly growing. The need of brief screening tools for problematic internet use in teenagers in Greece is imperative. The purpose of this study was to validate the 6-item short form of the Problematic Internet Use Questionnaire (PIUQ-SF-6) in a Greek sample of adolescents. The sample consisted of 200 adolescents (55.0% males, 61.6% high school students, 38.4% junior high school students) who completed the study’s questionnaires i.e., a demographic questionnaire, the PIUQ-SF-6, the Young’s Diagnostic Questionnaire (YDQ) and the Adolescent Computer Addiction Test (ACAT). The internal consistency of the Greek version of PIUQ-SF-6 was sufficient and acceptable (Cronbach’s alpha coefficient a = 0.80). Confirmatory Factor Analysis was significant, and goodness-of-fit was adequate. For establishing convergent validity, Pearson’s and Spearman’s correlation coefficients were calculated between the PIUQ-SF-6 and ACAT scales and Receiver Operating Characteristic (ROC) analysis (between PIUQ-SF-6 and YDQ) indicated an excellent accuracy. The Greek version of the PIUQ-SF-6 demonstrated satisfactory psychometric properties (reliability and validity) and is recommended as a reliable screening tool for problematic internet use in Greek adolescents.

## Introduction

Over the past decade the use of the internet has increased significantly and has become an integral part of our lives. Internet overuse is a global problem causing growing concern, which has led the scientific community to introduce terms such as “Problematic Internet Use” (PIU) or “Internet Addiction Disorder” (IAD). The term of IAD was first used by the New York based psychiatrist Ivan Goldberg (Dalal and Basu, 2002). The clinical psychologist Kimberly Young, in 1998, used the term “Addictive/ Pathological Internet use”, based on common features with abnormal gambling (Young, 1998).

Young *et al*. found that internet addiction covers a number of behaviours and impulse control problems and can be classified into five different types: (i) Cyber sexual addiction - compulsive use of adult websites for cybersex and cyber porn, (ii) Cyber-relationship addiction - over involvement in online relationships, (iii) Net compulsions - obsessive online gambling, shopping, or online trading, (iv) Information overload -compulsive web surfing or database search, (v) Computer addiction - obsessive computer game playing (Young *et al*., 2000). Davis RA distinguished two types of pathological internet use, general pathological use of the internet that includes a general, multidimensional excessive use of the internet, and specific pathological use of the internet that includes those cases of users who are addicted to a specific function or application of internet (pornographic material and services, games, online trading, auctions, gambling, etc.) (Davis, 2001).

Various studies on internet addiction have been conducted internationally. In South Korea, internet addiction has become one of the most serious public health problems following the deaths of 10 teenagers from cardiorespiratory diseases in internet cafes (Choi, 2007). More than 210,000 children and adolescents, aged 6–19 years, are addicted, with 80% of them in need of treatment and 20%−24% in need of hospitalisation. At the same time, since 2009, more than a thousand specialists have been trained in 190 mental health structures (Ahn, 2007). In Taiwan, Tsai and Lin (2003) found that among 700 adolescents, 12.8% were addicted to the internet (Tsai and Linn, 2003). In a study conducted on a Korean sample of 1,573 adolescents, it was found that 1.6% of respondents were addicted to the internet (Kyunghee *et al*., 2006). In Iran, among 1,968 adolescents, 3.8% were addicted (Gzassemzadeh, 2008). In Seoul, a survey of 903 adolescents showed that 10.7% were addicted and required further evaluation and intervention (Park *et al*., 2008).

In Greece, numerous studies have been conducted on adolescents’ computer addiction. One of the first studies in a random sample of 897 adolescents in the large urban center of the capital of Athens, found 12.8% of the adolescents with marginally healthy use of the internet, and only 1% with internet addiction. A key factor that determined the level of computer usage was the access points to it, as teenagers were found to spend more time online when at home or at internet cafes. In addition, users involved in social networking and video games showed a higher tendency to addiction (Tsitsika *et al*., 2009). A study in Thessaloniki, the second largest city in Greece, showed that in a sample of 278 high school students, 91% were aware that the use of social networks can lead to addiction, while only 9% claimed that the use of social networks cannot lead to addiction. Moreover, 55% of the sample were personally aware of cases of students who had become addicted to social networks (Samaras, 2014). In the Greek province, the problem of internet addiction seemed more intense, as a survey in Thessaly in a sample of 2,200 high school students reported internet addiction in 8.2% of users, mainly in boys who played online games and visited frequented internet cafes (Siomos *et al*., 2008). Karapetsas *et al*., in a survey conducted in Volos in a sample of adolescents aged 13 to 15 years, found an internet addiction rate of 22%, while gender was not found to be a determining factor (Karapetsas *et al*., 2012). A study conducted in the Greek island of Kos, in a sample of 1,270 adolescents aged 14–18 years, showed that 7.2% of boys and 5.1% of girls were addicted to the internet (Fisoun *et al*., 2012). A survey in adolescents aged 13 to 18 years, in the areas of Attica, Halkidiki and Rhodes in Greece, showed a 12% percentage of addicted students with higher rates of addiction in boys (15%) than girls (9%). Almost all students had a computer with internet connection at home, while those who did not have a computer visited internet cafes to access the internet (Sofos *et al*., 2011). Another study conducted in 2010 in a sample of students aged 11, 13 and 15 years, throughout Greece, found that 15.5% of the adolescents were addicted to the internet and 5.5% were addicted to electronic games. Specifically, the percentage of students who used the computer for at least 3 hours daily on weekdays, increased with age; from 14.1% at 11 years to 25.8% at 13 years and 33.7% at 15 years. In addition, more boys (26.8%) than girls (21.9%) used the computer for at least 3 hours daily on weekdays (Kokkevi *et al*., 2010).

Therefore, the need of brief screening tools for PIU in teenagers in Greece is imperative. The purpose of this study was to validate the 6-item short form of the Problematic Internet Use Questionnaire (PIUQ-SF-6) in a Greek sample of adolescents.

## Materials, Methodologies and Techniques

### Participants

The study was conducted from July to September 2020. The sample was recruited from three Greek cities; Athens (the urban capital of Greece with more than 5,000,000 residents), Nafplio (middle sized rural town with 33,356 citizens) and Nemea (small rural town with 6,483 citizens). The sample was set to be able to conduct factor analysis. According to the literature, the study sample should either follow the rule of 100, (Gorsuch, 1983; Kline, 1979), or five times higher than items (this means 30 subjects in the present study) (Hatcher, 1994), or at least 150 – 300 cases (Hutcheson and Sofroniou, 1999). Prior to study entry, students and their parents/guardians were asked to provide written consent.

### Materials

Participants were asked to declare few demographic characteristics such as sex, age, school class, parental educational level, and parental job status. Adolescents’ habits related to internet use were assessed with four questions about i) ownership or not of a personal computer (PC) they used, ii) hours spent online, iii) main reason of internet use, and iv) social media used. Adolescents were also asked to complete three questionnaires, the Problematic Internet Use Questionnaire-short form (PIUQ-SF-6), the Young’s Diagnostic Questionnaire (YDQ) and the Adolescent Computer Addiction Test (ACAT).

#### Problematic Internet Use Questionnaire (PIUQ):

The PIUQ comprises three versions (18-item, 9-item, and 6-item), all having reliable factor structures, and proven validity across both online and written data collection methods in samples of different age groups (*i.e*., adults and adolescents) (Demetrovics *et al*., 2016). For the basic outcome of the study, the validation of a short and quick instrument to measure problematic internet use, the 6-item short form of the PIUQ (PIUQ-SF-6) was used. The original version of the PIUQ with 18 items was developed on a Hungarian sample (Demetrovics, 2008). Years later, the short 9-item version (PIUQ-9) was developed (Laconi *et al*., 2019) with an aim to create a brief, comprehensive, non-arduous screening tool given that previous version caused fatigue and desertion. This version was evaluated in nine European samples of internet users that included 154 Greek adults. Another shorter version was developed using 6 items with an aim to assess more impulsive populations in limited time (Demetrovics *et al*., 2016). The 3-factor structure has been retained in all versions: obsession, neglect, and control disorder. A 5-point Likert scale (“never”, “rarely”, “sometimes”, “often”, “always/almost always”) was used to evaluate how much the given statements characterized the respondents. Scores range from 6 to 30, with higher scores indicating higher risk of PIU. In the original version of the PIUQ, Cronbach’s alpha was 0.87 (obsession α=0.85; neglect α=0.74; control disorder α=0.76) (Demetrovics, 2008). For the shorter version PIUQ-9, internal consistency ranged from 0.81 (German subsample) to 0.90 (Turkish subsample) (Laconi *et al*., 2019). For the 6-item version PIUQ-SF-6 Cronbach’s alpha was equal to α=0.77 (Demetrovics *et al*., 2019). The test–retest correlation of the original PIUQ was 0.90 (Demetrovics *et al*., 2008).

#### Validation procedure:

After obtaining permission from the developer to validate the PIUQ-SF-6 in a Greek population sample, the forward and back translation stage was carried out. Two bilingual translators, native speakers of Greek, translated the initial version of the PIUQ-SF-6 to the Greek language. They worked separately and each translator produced a written record of a translated version. Afterwards, the principal investigator unified the two translations into a single version since there were no significant differences between them. A third bilingual translator, native English speaker was recruited to translate backward the Greek version to the English language, without having ever read the original questionnaire. A three-person expert committee (including the principal investigator, a professor and a statistician experienced in cultural adaptation) supervised the translated versions and finalised the Greek version of the PIUQ-SF-6.

A pilot test in 15 participants was conducted to identify any confusing questions or meanings as well as suggestions for possible improvement. Moreover, during the pilot test the principal instigator counted the time needed for completion of the questionnaire (approximately 3 minutes). Reliability analysis, and more specifically Cronbach’s alpha, was carried out in this phase to examine the internal consistency of the PIUQ-SF-6 which was found equal to a = 0.72, an acceptable value as it was higher than the threshold of 0.7 (De Vellis, 1991).

#### Young’s Diagnostic Questionnaire (YDQ):

For convergent validity and for Receiver Operating Characteristic (ROC) analysis the Young’s Diagnostic Questionnaire (Young, 1996) was used, which is validated in the Greek population (Siomos *et al*., 2008). Young developed the YDQ from the pathological gambling DSM-IV criteria, using seven of ten DSM criteria and adding an eighth item (Young, 1996; Siomos *et al*., 2008). The YDQ consists of eight nominal questions (yes-or-no) regarding internet use. Participants who answer positively to five or more of the eight items are classified as addicted internet users, while the remaining participants are classified as normal internet users. The consistency of the Greek version of the YDQ was tested with Cronbach’s alpha which was a = 0.72 (Siomos *et al*., 2008).

#### Adolescent Computer Addiction Test (ACAT):

A second tool used for convergent validity was the Adolescent Computer Addiction Test which was validated in Greek students in 2006 (Siomos *et al*., 2009) The ACAT is a 20-question tool with 5-point Likert type possible answers (1, not at all; 2, rarely; 3, occasionally; 4, often; 5, always). After scoring the tool, four scales were constructed: Computer Addiction, Work Neglect, Social Life Neglect, Extreme Use. The ACAT has excellent test-retest reliability, internal consistency, and construct validity; the total scale reliability was a = 0.93, and the range for four scales was a = 0.74 to a = 0.85 (Siomos *et al*., 2009).

### Statistical analysis

Absolute and relative frequencies were used to describe categorical variables such as demographic characteristics. Mean (M), standard deviation (SD) and median (Mdn) described continuous data. The internal consistency was carried out using Cronbach’s alpha; values above 0.7 were considered acceptable (Cortina, 1993). Exploratory and Confirmatory Factor Analysis (EFA and CFA, respectively) were performed to examine the validated tool. For EFA, Varimax rotation was used. For CFA, chi-square test (χ^2^), comparative fit index (CFI), Tucker-Lewis index (TLI), root mean square error of approximation (RMSEA) and standardized root mean residual (SRMR) were computed to evaluate the model. The model can be considered satisfactory when the CFI and TLI values are higher than or close to 0.95 and still acceptable if the values are above 0.90 (Brown, 2006; Bentler and Bonett, 1980). An RMSEA value below 0.05 indicates excellent fit, a value around 0.08 indicates adequate fit, a value above 0.10 indicates poor fit and SRMR values below 0.10 indicate good fit (Brown, 2006; Browne and Cudeck, 1993). To check the convergent validity, Pearson’s and Spearman rho correlation coefficients were computed. Convergent validity was considered an adequate instrument measuring the same construct when the correlation between PIUQ-SF-6 and ACAT was >0.50 (Abma *et al*., 2016). Also, ROC analysis and Area Under Curve (AUC) were established to check convergent validity as well (0.7 to 0.8 is considered acceptable, 0.8 to 0.9 is considered excellent, and more than 0.9 is considered outstanding) (Hosmer and Lemeshow, 2000).

Statistical analysis was performed using the SPSS version 25.0 (IBM Corp. Released 2017. IBM SPSS Statistics for Windows, Version 25.0. Armonk, NY: IBM Corp) and R Statistics software version 4.0.3 (R Core Team, 2020), and the ROC curve was fit and analysed using the Lavaan package (Rosseel, 2012).

## Results

A total of 200 adolescents aged 12–17 years completed the questionnaires. Participants’ demographic and other characteristics are presented in Table 1.

Specifically, 55.0% (n = 110) of the respondents were male, while females constituted 45.0% of the sample (n = 90). Most students were in high school (61.6%) while 38.4% were in junior high school. Almost 8 out of 10 participants had parents who were high school graduates or had a University degree. Almost 10 out of 10 fathers were employed, whereas 8 out of 10 mothers were in employment. Nine in 10 students had a personal computer at home (25.9% reported exclusive possession and 75.6% shared use with other family members). Regarding the hours they spent on the internet on a daily basis, 17.2% of the participants spent 1–2 hours, 37.9% 2–3 hours, 34.8% 3–4 hours, 8.6% more than 4 hours and the rest 1.5% did not own a computer and did not use internet at all. The main reasons for using internet were to chat with friends or other people (37.9%) and to play games (33.8%). The most popular social media were Facebook (32.6%), followed by Instagram (25.5%) and Viber/WhatsApp (23.4%).

### Exploratory Factor analysis:

The EFA of PIUQ-SF-6 is presented in Table 2. Factor analysis showed good adaptability in the Obsession scale and mild adaptability in the Neglect and Control Disorder scales. The total variance explained by EFA was 76.27%. The mean, median and standard deviation for Obsession were 2.19, 2.00 and 0.78, for Neglect 2.51, 2.50 and 0.88, and for Control Disorder 2.25, 2.00 and 0.78, respectively.

Regarding the internal consistency of the Greek version of the PIUQ-SF-6, Cronbach’s alpha coefficient was a = 0.80 for the total scale (Table 2).

### Confirmatory Factor analysis:

The CFA of the PIUQ-SF-6 is presented in Table 3. Baseline model was found significant (χ^2^ = 358.45, p < 0.001). The goodness-of-fit summary was adequate; TLI = 0.88; CFI = 0.95; SRMR = 0.04. The only index that was not good enough is RMSEA which was 0.12, slightly higher than 0.10.

### Convergent validity:

To prove convergent validity, Pearson’s and Spearman’s correlation coefficients between the PIUQ-SF-6 and ACAT scales were calculated (Table 4).

Strongest correlations were found between Control Disorder and Extreme Use (r = 0.701, p < 0.001), Neglect and Extreme Use (r = 0.682, p < 0.001), Obsession and Computer Addiction (r = 0.381, p < 0.001).

To measure convergent validity, ROC analysis between PIUQ-SF-6 and YDQ was carried out (Table 5). For the total scale of PIUQ-SF-6, Control Disorder, and Obsession scale results indicated an excellent accuracy (AUC = 0.81, p < 0.001, 95% CI [0.74, 0.88], AUC = 0.82, p < 0.001, 95% CI [0.75, 0.88] and AUC = 0.78, p < 0.001, 95% CI [0.70, 0.86] respectively). For the Neglect scale, results indicated an acceptable accuracy (AUC = 0.67, p = 0.001, 95% CI [0.59, 0.75]).

## Discussion

Internet is a means of communication, entertainment and education that is constantly evolving, exerting a significant influence on the shaping of modern reality. While internet is a necessary tool of everyday routine, the concern for development of pathological behaviours in relation to its use is constantly growing. The negative effects on young people’s physical health, due to the extensive screen time and sedentary lifestyle, internet addiction, excessive involvement with online games and social networking sites are of particular concern to the scientific community. In Greece, digital literacy has not come through a coordinated learning process but through commercial engagement, resulting in high levels of internet addiction (Sfakianakis *et al*., 2012).

The PIUQ-SF-6 is a comprehensive, brief tool that assesses three key factors in PIU; obsession (*i.e*., obsessive thinking about the internet and mental withdrawal symptoms caused by the lack of internet use), neglect (*i.e*., neglect of basic needs and everyday activities), and control disorder (*i.e*., difficulties in controlling internet use).

The basic criterion for the reliability of a scale is the Cronbach’s alpha coefficient with acceptable values greater than 0.7 (DeVellis, 1991). With regards to the validation of PIUQ-SF-6, the questionnaire was checked for its internal consistency and was found sufficient and acceptable. For the initial version of the PIUQ-18, the Cronbach alpha was equal to 0.87, while for the smaller version of the PIUQ-9, the internal consistency was 0.81 in the German sample and 0.90 in the Turkish sample. In our study, the CFI index was 0.95 and the SRMR index was 0.04. Furthermore, in the initial version of PIUQ-18, the questionnaire contains 6 questions per factor, while in the PIUQ-9 version the questionnaire contains 3 questions per factor, and in the smaller PIUQ-SF-6 version it contains 2 questions per factor.

The main limitation of the study was that the sample was not representative of all areas in Greece, rural and urban. In addition, the study sample was relatively small compared to that of the original PIUQ-SF-6 questionnaire survey (n=5,005) (Demetrovics, 2016).

To conclude, the Greek version of the PIUQ-SF-6 demonstrated satisfactory psychometric properties (reliability and validity) and is recommended as a reliable screening tool for PIU in Greek adolescents. Future large surveys are needed to provide more in-depth knowledge about PIU in children and adolescents in Greece.

## Figures and Tables

**Table 1.** Demographic characteristics of the sample (N = 200).

Characteristic		Absolute frequency	Relative frequency (%)
**Sex**	Male	110	55.0
	Female	90	45.0
**School Class**	Junior high school	76	38.4
	High school	122	61.6
**Paternal educational level**	Junior high school	1	0.5
	High school	80	40.4
	College/Trade school	16	8.1
	University degree	96	48.5
	Master/PhD	5	2.5
**Maternal educational level**	Junior high school	5	2.5
	High school	68	34.3
	College/Trade school	26	13.1
	University degree	91	46.0
	Master/PhD	8	4.0
**Does your father have a job?**	Yes	196	99.5
	No	1	0.5
**Does your mother have a job?**	Yes	155	78.3
	No	43	21.7
**The Personal Computer that you use:**	Exclusively mine	51	25.9
	Shared with other family members	143	72.6
	I do not use at home	3	1.5
**How many hours a day do you use the internet?**	1–2 hours	34	17.2
	2–3 hours	75	37.9
	3–4 hours	69	34.8
	More than 4 hours	17	8.6
	I do not use at all	3	1.5
**What is the main reason you use Internet?**	To play videogames	66	33.8
	To find information about homework	22	11.3
	To chat with friends or other people	74	37.9
	For online shopping	3	1.5
	To download movies or songs	24	12.3
	To be informed	6	3.1
**Social media that you use (multiple choice question)**	Facebook	180	32.6
	Instagram	141	25.5
	Snapchat	75	13.6
	Viber/WhatsApp	129	23.4
	Twitter	12	2.2
	TikTok	15	2.7

**Table 2.** Factor loadings and descriptive statistics of three factors of the PIUQ-SF-6.

	Obsession	Neglect	Control
**Disorder**			
**1. How often do you spend time online when you would rather sleep?**		0.889	
**2. How often do you feel tense, irritated, or stressed if you cannot use the Internet for as long as you want to?**	0.916		
**3. How often does it happen to you that you wish to decrease the amount of time spent online but you do not succeed?**		0.365	0.640
**4. How often do you try to conceal the amount of time spent online?**			0.781
**5. How often do people in your life complain about spending too much time online?**		0.774	0.373
**6. How often does it happen to you that you feel depressed, moody, or nervous when you are not on the Internet and these feelings stop once you are back online?**	0.689		0.587
**Eigenvalue**	1.71	1.44	1.43
**Variance explained (%)**	28.53	23.99	23.75
**Mean**	2.19	2.51	2.25
**Median**	2.00	2.50	2.00
**Standard deviation**	0.78	0.88	0.78

Values refer to loading after Varimax rotation, eigenvalue, percent of variance explained, mean, median, standard deviation.

Cronbach’s alpha of the PIUQ-SF-6 was α=0.80.

PIUQ-SF-6, Problematic Internet Use Questionnaire-Short Form (6-item)

**Table 3.** Confirmatory Factor Analysis of the PIUQ-SF-6.

Latent Variables	Latent Variables	Estimate	Std. Error	z-value	p
Obsession	Q2	0.59	0.06	9.49	<0.001
	Q6	0.76	0.06	12.62	<0.001
Neglect	Q1	0.54	0.07	7.26	<0.001
	Q5	0.83	0.09	9.43	<0.001
Control Disorder	Q3	0.53	0.08	6.77	<0.001
	Q4	0.56	0.07	7.84	<0.001
Chi-square	358.45				
Df	15				
P	<0.001				
CFI	0.95				
TLI	0.88				
RMSEA [95%CI]	0.12 [0.07, 0.17]				
SRMR	0.04				
AIC	2972.26				
BIC	3021.66				
SSABIC	2974.14				

Q1 to Q6, questions of PIUQ-SF-6 as referred in Table 2.

PIUQ-SF-6, Problematic Internet Use Questionnaire-Short Form (6-item); df, degrees of freedom; CFI, Comparative Fit Index; TLI, Ticker-Lewis Index; RMSEA; Root Mean Square Error of Approximation; 95% CI, 95% Confidence Interval; SRMR, Standardized Root Mean Square Residual; AIC, Akaike Information Criteria; BIC, Bayesian Information Criteria; SSABIC, sample size adjusted Bayesian Information Criteria.

**Table 4.** Correlations between PIUQ-SF-6 and ACAT scales.

	1	2	3	4	5	6	7
**1.Obsession§**	1	0.491***	0.574***	0.681***	0.609***	0.624***	0.570***
**2. Neglect**		1	0.608***	0.514***	0.510***	0.438***	0.682***
**3. Control Disorder**			1	0.675***	0.664***	0.617***	0.701***
**4. Computer Addiction**				1	0.776***	0.712***	0.713***
**5. Work neglect**					1	0.718***	0.641***
**6. Social life neglect**						1	0.564***
**7. Extreme use**							1

*** p< 0.001;

** p< 0.01;

* p< 0.05 (Pearson, §Spearman rho correlation coefficients).

PIUQ-SF-6, Problematic Internet Use Questionnaire-Short Form (6-item); ACAT, Adolescent Computer Addiction Test

**Table 5.** ROC analysis and AUC between PIUQ-SF-6 and YDQ.

Test Result Variables	Area	Std. Error^a^	Asymptotic p-value^b^	Asymptotic 95% Confidence Interval	
				Lower Bound	Upper Bound
**Obsession**	0.78	0.04	<0.001	0.70	0.86
**Neglect**	0.67	0.04	0.001	0.59	0.75
**Control Disorder**	0.82	0.03	<0.001	0.75	0.88
**PIUQ-SF-6 Total Scale**	0.81	0.03	<0.001	0.74	0.88

The test result variables: Obsession, Neglect, Control Disorder, PIUQ-SF-6.

State variable: Addicted Internet users according to YDQ.

a. Under the nonparametric assumption

b. Null hypothesis: true area = 0.5

ROC, Receiver Operating Characteristic; AUC, Area Under Curve; PIUQ-SF-6, Problematic Internet Use Questionnaire-Short Form (6-item); YDQ, Young’s Diagnostic Questionnaire

## Key Points

- Problematic Internet Use (PIU) is prevalent among teenagers.
- The need of brief screening tools for PIU in adolescents is imperative.
- The Greek version of the PIU Questionnaire-short form (PIUQ-SF-6) has satisfactory reliability and validity.
- The PIUQ-SF-6 is a reliable screening tool for PIU in Greek adolescents.
